# Serum myo-inositol oxygenase levels at hospital discharge predict progression to chronic kidney disease in community-acquired acute kidney injury

**DOI:** 10.1038/s41598-022-17599-w

**Published:** 2022-08-02

**Authors:** Tom Jose Kakkanattu, Jaskiran Kaur, Vinod Nagesh, Monica Kundu, Kajal Kamboj, Prabhjot Kaur, Jasmine Sethi, Harbir Singh Kohli, Kishan Lal Gupta, Arpita Ghosh, Vivek Kumar, Ashok Kumar Yadav, Vivekanand Jha

**Affiliations:** 1 Department of Nephrology, Postgraduate Institute of Medical Institute Education and Research, Chandigarh, 160012 India; 2 Department of Experimental Medicine and Biotechnology, Postgraduate Institute of Medical Institute Education and Research, Chandigarh, 160012 India; 3 George Institute for Global Health, UNSW, New Delhi, India; 4 School of Public Health, Imperial College, London, UK; 5 Manipal Academy of Higher Education, Manipal, India

**Keywords:** Kidney diseases, Acute kidney injury

## Abstract

Acute kidney injury (AKI) increases the risk of morbidity, mortality, and progression to chronic kidney disease (CKD). There are few data on the risk of CKD following community-acquired AKI (CA-AKI) and its predictors from developing countries. We evaluated the association of a panel of serum and urine biomarkers at the time of hospital discharge with 4-month renal outcome in CA-AKI. Patients of either sex, aged between 18 and 70 years, with no underlying CKD, and with CA-AKI were recruited at the time of discharge from hospital in this prospective observational study. Levels of serum and urine biomarkers were analyzed and association between these markers and development of CKD, defined as eGFR < 60 ml/min/1.73 m^2^ or dialysis dependence at 4 month after discharge, were analyzed using multivariate logistic regression analysis and penalized least absolute shrinkage and selection operator logistic regression. Out of a total 126 patients followed up for 4 months, 25 developed CKD. Those who developed CKD were older (p = 0.008), had higher serum creatinine (p < 0.001) and lower serum albumin (p = 0.001) at discharge. Adjusted logistic regression showed that each 10% increase in standardized serum myo-inositol oxygenase (MIOX) level increased the odds of progression to CKD by 13.5%. With 10% increase in standardized urine Neutrophil gelatinase-associated lipocalin (NGAL), serum creatinine and urine protein creatinine ratio (uPCR), increase in the odds of progression to CKD was 10.5%, 9.6% and 8%, respectively. Multivariable logistic model including serum MIOX, discharge serum creatinine and discharge uPCR, was able to predict the progression of CKD [AUC ROC 0.88; (95% CI 0.81, 0.95)]. High level serum MIOX levels at the time of discharge from hospital are associated with progression to CKD in patients with CA-AKI.

## Introduction

Acute kidney injury (AKI) is a rising global health concern. In developing countries, especially in the in rural areas, AKI frequently develops in community settings in association with infections, envenomation, diarrhea, and is generally more common in the otherwise healthy population^1^.

Existing literature suggests the role of AKI in increasing the risk of morbidity, mortality, and progression to chronic kidney disease (CKD)^2,3^. Patients with AKI who had incomplete or non-recovery, had an increased risk of mortality and adverse renal outcomes when compared with fully recovered patients^4^. The ability to forecast renal recovery in an individual patient will help in clinical decision-making and facilitate clinical research in this field. If the patients who survive AKI and are at an increased risk for progression to CKD can be identified, they can be subjected to better follow-up and their natural history might be impacted favorably^5^. Improved understanding of pathophysiological mechanism of the worsening of AKI could help in developing novel medications that can hamper the progression of AKI and thus improve the long-term outcome of AKI.

Serum creatinine, the currently used marker for AKI, lacks sensitivity and is affected by a number of factors including muscle mass, fluid balance during ICU stay, and decreased production during critical illness^6,7^. Robust research is ongoing to identify biomarkers for risk assessment, early detection of AKI, prognostication, and prediction of the chance of progression to CKD. The 23rd Acute Disease Quality Initiative Consensus Conference recommends that the biomarkers predictive of CKD progression should be incorporated into a comprehensive post-AKI/acute kidney disease (AKD) care bundle (Grade C recommendation)^8^. They also suggest that a combination of damage and functional biomarkers, along with clinical information be used to improve patient care and outcomes (Grade C recommendation)^8^. The FROG-ICU study in 1207 intensive care unit (ICU) patients showed that abnormal values of plasma and urine neutrophil gelatinase-associated lipocalin (NGAL), plasma proenkephalin A and plasma cystatin C at hospital discharge were predictive of higher mortality at 1-year^9^. Neutrophil gelatinase-associated lipocalin (NGAL), Kidney injury molecule (KIM-1), Liver-type fatty acid-binding protein(L-FABP), Myoinositol oxygenase (MIOX) and Yes-associated protein (YAP) are emerging as important biomarkers for early detection of AKI in various settings and would be logical choices to evaluate for prediction of recovery from AKI^10–15^. New interest has been seen in the field of Nicotinamide adenine dinucleotide (NAD+) biosynthesis. Urinary quinolinate /tryptophan ratio(uQ/T), indicative of reduced quinolinate phosphoribosyltransferase (QPRT), an enzyme involved in NAD+ biosynthesis, was found to predict AKI and other adverse outcomes in animal studies and in critically ill patients^16^.

There is little research, however in the utility of these biomarkers in community-acquired AKI (CA-AKI) in developing countries. In this study, we evaluated the association of a panel of serum and urine biomarkers (MIOX, YAP, NGAL, KIM-1, LFABP1 and urinary quinolinate/tryptophan ratio) at the time of hospital discharge with the renal outcomes at 4 months in CA-AKI.

## Results

**Figure 1:**
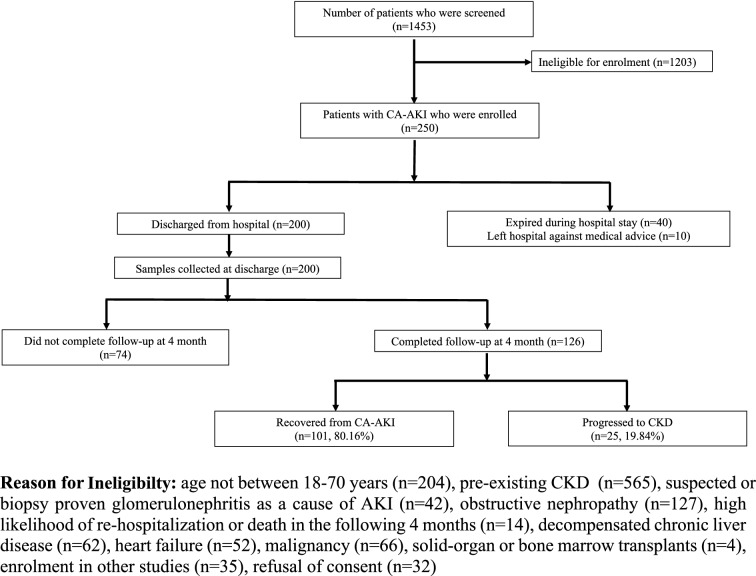
Study consort flowchart.

A total of 1,453 patients with a diagnosis of AKI were screened between February 2017 and January 2020. After excluding 1203 patients, 250 patients were enrolled. A total of 124 patients died or were lost to follow-up, leaving 126 patients for the final analysis (Fig. 1). The mean age of study participants was 37.23 ± 15.20 years and 43.7% were females. 93.7% of patients were in stage 3 of AKI. The main cause of CA-AKI was infection (47%) followed by obstetric (16%), envenomation (14%), over the counter or indigenous drug related (7%), and other causes (16%). Infections included acute gastroenteritis (37%), undifferentiated acute febrile illness (27%), urinary tract infection (10%), scrub typhus (9%), leptospirosis (5%), malaria (5%) and others (7%). Out of 126 patients, progression to CKD at 4 months was observed in 19.84% (n = 25).

The patient characteristics including laboratory investigations are shown in Tables 1 and 2. Patients who progressed to CKD were older (44.40 ± 15.32 vs 35.46 ± 14.72 years, p = 0.008), had longer duration of hospitalization (16.40 ± 9.02 vs 12.29 ± 9.45 days, p = 0.052), and had higher serum creatinine (p < 0.001), and lower serum albumin at discharge (p = 0.001). Further, uPCR at the time of hospital discharge was higher in these patients as compared to recovered patients [896 (317, 2128) vs 232 (131, 588) mg/gm, p < 0.001].

**Table 1:** Baseline patient characteristics in the study participants.

Parameter (s)	All cases	Recovered from CA-AKI (n = 101)	Progressed to CKD (n = 25)	P value*
Age (years)	37.23 ± 15.20	35.46 ± 14.72	44.40 ± 15.32	0.008
Female sex	55 (43.7%)	44 (43.6%)	11 (44.0%)	0.969
**Comorbidities**				
Diabetes	5 (4%)	3 (3.0%)	2 (8%)	0.561
Hypertension	14 (11.1%)	8 (7.9%)	6 (24.0%)	0.022
**qSOFA score**				
GCS (< 15 = 1), score of 1	1 (0.8%)	1 (1%)	0 (0%)	1.0
SBP (< 100 mmHg = 1), score of 1	61 (48.4%)	50 (49.5%)	11 (44%)	0.622
RR (> 22/min = 1), score of 1	51 (40.5%)	35 (34.7%)	16 (64%)	0.007
**Total qSOFA score**				
0	43 (34.1%)	37 (36.6%)	6 (24%)	0.391
1	53 (42.1%)	42 (41.6%)	11 (44%)	
2	30 (23.8%)	22 (21.8%)	8 (32%)	
Oliguria during admission	114 (90.5%)	91 (90.1%)	23 (92%)	1.0
Maximum creatinine at admission(mg/dL)	7.05 ± 2.81	6.87 ± 2.70	7.76 ± 3.19	0.158
**AKI stage**				
Stage 2	8 (6.3%)	6 (5.9%)	2 (8%)	0.658
Stage 3	118 (93.7%)	95 (94.1%)	23 (92%)	
Requirement of KRT	93 (73.8%)	71 (70.3%)	22 (88%)	0.081
Duration of hospitalization (days)	13.13 ± 9.47	12.29 ± 9.45	16.40 ± 9.02	0.052

Data presented as mean ± standard deviation and number (percentage).

*p-value for comparison between patients recovered and progressed to CKD from CA-AKI.

*qSOFA* Quick sequential organ failure assessment score, *GCS* Glasgow coma scale, *SBP* Systolic blood pressure, *RR* Respiratory rate, *KRT* Kidney replacement therapy (all were haemodialysis).

**Table 2:** Clinical characteristics, serum and urine biomarkers at the time of discharge in study subjects.

Parameter median	All cases (n = 126)	Recovered from CA-AKI (n = 101)	Progressed to CKD (n = 25)	P values*
Hemoglobin (g/dL)	10.03 ± 2.02	10.21 ± 2.05	9.30 ± 1.72	0.035
Serum urea (mg/dL)	47.00 (30.40, 81.60)	55.20 (29.15, 76.13)	72.33 (49.21, 117.00)	0.002
Serum creatinine (mg/dL)	2.20 (1.21, 4.05)	1.8 (1.10, 3.90)	3.6 (2.51, 6.30)	< 0.001
Serum albumin (g/dL)	3.48 ± 0.78	3.60 ± 0.78	3.02 ± 0.63	0.001
Spot uPCR (mg/gm)	287 (141, 757)	232 (131, 588)	896 (317, 2128)	< 0.001
Serum MIOX (pg/mL)	3222 (2299, 4435)	3005 (2221, 3807)	4995 (3280, 6107)	< 0.001
Serum YAP (pg/mL)	903 (333, 1721)	931 (304,1553)	718 (364,1867)	0.833
Urine MIOX/creatinine (pg/g)	1540 (914, 2400)	1547 (910, 2342)	1446 (867, 3014)	0.805
Urine YAP/creatinine (ng/g)	304 (195, 516)	307 (195, 481)	272 (201, 747)	0.647
Urine KIM-1/creatinine (pg/g)	9.59 (2.10, 305.11)	7.05 (1.91, 306.13)	25.55 (3.42, 365.70)	0.367
Urine NGAL/creatinine (ng/g)	56.08 (18.88,140.06)	44.93 (15.32, 116.14)	159.75 (51.26, 357.69)	< 0.001
Urine L-FABP/creatinine (ng/g)	88.21 (6.84, 775.10)	274.79 (7.50, 911.97)	10.98 (5.24, 287.11)	0.035
Urine quinolinate/tryptophan (uQ/T)	1.52 (0.61, 4.87)	1.40 (0.61, 4.83)	2.73 (0.42, 7.82)	0.592

Data presented as mean ± standard deviation and median (25th,75th, percentile).

*p-value for comparison between patients recovered and progressed to CKD from CA-AKI.

*uPCR* urine protein creatinine ratio, *MIOX* myo-inositol oxygenase, *YAP* Yes-associated protein, *KIM-1* kidney injury molecule-1. *NGAL* neutrophil gelatinase-associated lipocalin, *L-FABP* liver-type fatty acid binding protein.

### Biomarkers and recovery from CA-AKI

**Figure 2:**
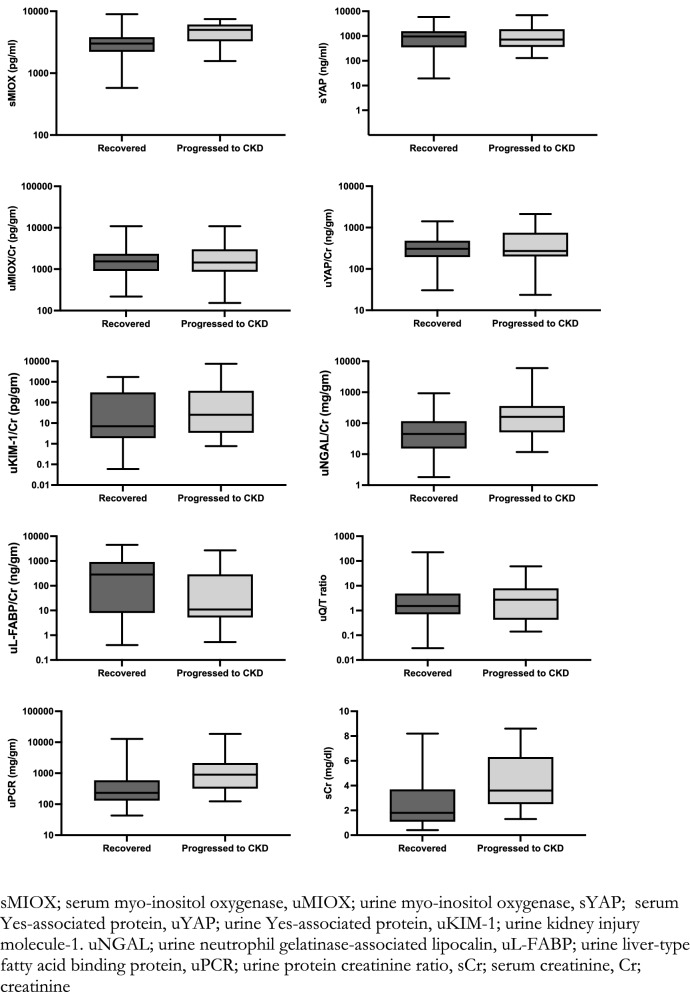
Box and whisker plot showing the levels of serum and urine biomarkers in recovered patients and those who progressed to CKD from CA-AKI. *sMIOX* serum myo-inositol oxygenase, *uMIOX* urine myo-inositol oxygenase, *sYAP* serum Yes-associated protein, *uYAP* urine Yes-associated protein, *uKIM-1* urine kidney injury molecule-1, *uNGAL* urine neutrophil gelatinase-associated lipocalin, *uL-FABP* urine liver-type fatty acid binding protein, *uPCR* urine protein creatinine ratio, *sCr* serum creatinine, Cr; creatinine.

The serum and urine biomarkers at the time of hospital discharge have been tabulated in Table 2 and Fig. 2. Urine biomarker values were normalized using spot urine creatinine concentration. Serum MIOX was higher in patient who progressed to CKD [4995 (3280, 6107) vs 3005 (2221, 3807) pg/ml, p < 0.001]. High level of urine NGAL/creatinine [159.75 (51.26, 357.69) vs 44.93 (15.32, 116.14) ng/g, p < 0.001] and uPCR [896 (317, 2128) vs 232 (131, 588) mg/g, p < 0.001] whereas low urine LFABP/creatinine [10.98 (5.24, 287.11) vs 274.79 (7.50, 911.97) ng/g, p = 0.035] was also noted in these subjects as compared to patient who recovered from AKI. There was no statistically significant difference between groups with regards to urinary MIOX level, serum and urinary YAP levels, urine KIM-1 and quinolinate to tryptophan ratio (uQ/T) (Table 2).

Unadjusted logistic regression analysis showed that a 10% increase in standardized serum MIOX level increased the odds of progression to CKD by 12.8% [OR 3.58, 95% CI (1.84, 6.96), p < 0.001]. With every 10% increase in standardized urine NGAL [OR 2.69, 95% CI (1.56, 4.63), p < 0.001], and standardized serum creatinine [OR 2.69 95% CI (1.54, 4.69), p < 0.001], the odds of progression to CKD increased by 9.8%. For 10% increase in standardized uPCR [OR 2.61 95% CI (1.61, 4.23), p < 0.001] level, odds of progression to CKD rose by 9.5%. (Table 3).

**Table 3:** Unadjusted logistic regression of clinical parameters, serum and urine biomarkers at hospital discharge and for the association of renal outcome (progression to CKD from CA-AKI).

Parameter	OR (95% CI)	Percent change with respect to 10% change in level of biomarkers*	P value
Age (years)	1.04 (1.01–1.07)	–	0.011
Sex Male Female	1.02 (0.42, 2.46)	–	0.969
Diabetes No Yes	2.84 (0.45, 17.99)	–	0.268
Hypertension No Yes	3.67 (1.14, 11.80)	–	0.029
RRT (no) yes	3.10 (0.86, 11.14)	–	0.083
**qSOFA score (0)**		–	
1	1.62 (0.54, 4.80)		0.388
2	2.24 (0.69, 7.32)		0.181
Discharge creatinine (mg/dL)	2.69 (1.54, 4.69)	9.8	< 0.001
Spot uPCR (mg/g)	2.61 (1.61, 4.23)	9.5	< 0.001
Serum MiOX (pg/mL)	3.58 (1.84, 6.96)	12.8	< 0.001
Serum YAP (pg/mL)	1.18 (0.70, 1.96)	1.5	0.534
Urine MIOX/creatinine (pg/g)	1.08 (0.67, 1.73)	0.7	0.759
Urine YAP/creatinine (ng/g)	1.11 (0.69, 1.80)	0.9	0.668
Urine KIM-1/creatinine (pg/g)	1.25 (0.80, 1.95)	2.1	0.321
Urine NGAL/creatinine (ng/g)	2.69 (1.56, 4.63)	9.8	< 0.001
Urine L-FABP/creatinine (ng/g)	0.68 (0.43, 1.06)	− 3.6	0.090
Urine quinolinate/tryptophan (uQ/T)	1.11 (0.69, 1.81)	0.9	0.664

Continuous variables except for age, are standardized log transformed.

*uPCR* urine protein creatinine ratio, *MIOX* myo-inositol oxygenase, *YAP* Yes-associated protein, *KIM-1* kidney injury molecule-1. *NGAL* neutrophil gelatinase-associated lipocalin, *L-FABP* liver-type fatty acid binding protein.

*Percentage change with respect to 10% change in levels of biomarkers is represented for the standardized log transformed variable computed from odds ratio (OR) by formula; 100 ×(exp(log(OR) × log(1.1)) − 1).

After adjusting with age, sex, hypertension, diabetes, requirement of kidney replacement therapy (KRT), quick sequential organ failure assessment (qSOFA) score, and duration of hospitalization; serum MIOX, urinary NGAL, serum creatinine and uPCR were independently associated with progression to CKD (Table 4). 10% increase in standardized serum MIOX level increased the odds of progression to CKD by 13.5% [OR 3.81, 95% CI (1.66, 8.73)]. With 10% increase in standardized urine NGAL level [OR 2.88, 95% CI (1.39, 5.99)], and standardized serum creatinine level [OR 2.63, 95% CI (1.30, 5.31)], increase in the odds of progression to CKD was 10.5% and 9.6%, respectively. For standardized uPCR [OR 2.27, 95% CI (1.29, 3.98)], 10% increase in its level would rise odds of progression to CKD by 8%.

**Table 4:** Adjusted logistic regression of serum and urine biomarkers at hospital discharge for the association of renal outcome (progression to CKD from CA-AKI).

Parameter	OR (95% CI)	Percent change with respect to 10% change in level of biomarkers*	P value
Serum MIOX (pg/mL)	3.81 (1.66, 8.73)	13.5	0.002
Serum YAP (pg/mL)	1.12 (0.59, 2.13)	1.0	0.736
Urine MIOX/creatinine (pg/g)	0.99 (0.56, 1.75)	− 0.1	0.971
Urine YAP/creatinine (ng/g)	0.97 (0.55, 1.72)	− 0.3	0.910
Urine KIM-1/creatinine (pg/g)	1.44 (0.83, 2.51)	3.5	0.192
Urine NGAL/creatinine (ng/g)	2.88 (1.39, 5.99)	10.5	0.005
Urine L-FABP/creatinine (ng/g)	0.66 (0.38, 1.15)	− 3.9	0.142
Urine quinolinate/tryptophan (uQ/T)	1.32 (0.73, 2.38)	2.6	0.362
Serum creatinine (mg/dL)	2.63 (1.30, 5.31)	9.6	0.007
Spot uPCR (mg/g)	2.27 (1.29, 3.98)	8.0	0.004

Adjusted variables included age, sex, q-SOFA score, comorbidities (diabetes and hypertension), KRT and duration of hospitalization.

*uPCR* urine protein creatinine ratio, *MIOX* myo-inositol oxygenase, *YAP* Yes-associated protein, *KIM-1* kidney injury molecule-1. *NGAL* neutrophil gelatinase-associated lipocalin, *L-FABP* liver-type fatty acid binding protein.

*Percentage change with respect to 10% change in levels of biomarkers, is represented as these variables are standardized log transformed. It was computed from odds ratio (OR) by formula; 100 ×(exp(log(OR) × log(1.1)) − 1).

**Figure 3:**
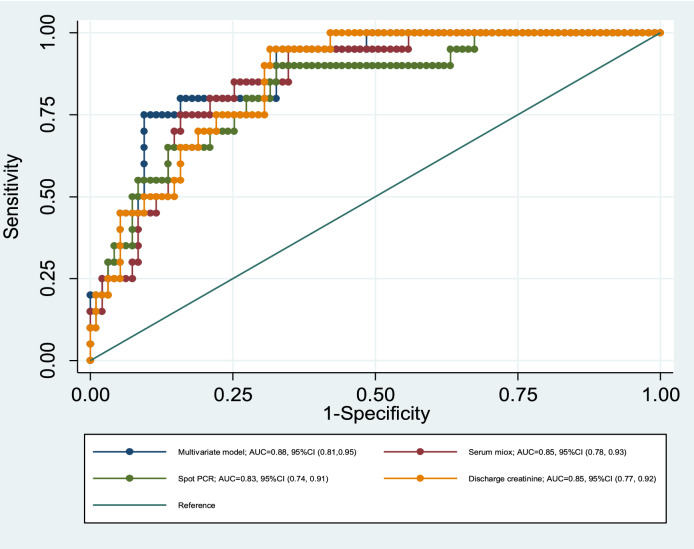
Receiver operating characteristic curves (ROC) for the multivariate model and its individual constituents. *AUC* area under the curve.

Penalized LASSO logistic regression with AKI to CKD progression as the outcome and all serum and urine biomarkers plus other parameters including age, sex, diabetes, hypertension, qSOFA score, requirement of KRT, and hospital duration as potential predictors resulted in a final model containing age, sex, hypertension, hospital duration, serum MIOX, discharge uPCR, and discharge serum creatinine. Linear predictor values for all study subjects were calculated using the intercept and coefficients from the multivariable model (shown in Supplementary Table 1). Finally, the logistic regression model was fit for only the select variables obtained from penalized LASSO regression to assess the association with progression to CKD. The ROC-AUC of the multivariable logistic model with selected parameters was 0.88; (95% CI 0.81, 0.95) and was significantly greater than those of any of the 3 variables analysed individually; serum MIOX (AUC 0.85; 95% CI 0.78, 0.93), discharge serum creatinine (AUC = 0.85; 95% CI = 0.87, 0.92) and discharge uPCR (AUC 0.83; 95% CI 0.74, 0.91) (Fig. 3).

## Discussion

In this first of its kind prospective cohort study of patients with CA-AKI from a developing country, we show that serum MIOX, urine NGAL, serum creatinine and uPCR at the time of discharge were independently associated with progression to CKD from CA-AKI after adjusting for gender, age, hypertension, diabetes, requirement of renal replacement therapy and duration of hospitalization. Further, in multivariable model using penalized LASSO regression with selected variables, serum MIOX, serum creatinine and uPCR remained independent predictors of CKD progression from CA-AKI and multivariable model including these three markers performed better as compared to individual markers in LASSO regression analysis.

In the panel of analyzed biomarkers, serum MIOX was the only one that remained independent predictor of CKD progression from CA-AKI along after multivariable regression analysis as well as in the multivariable predictive model. MIOX is a monomeric renal-specific proximal tubule protein that oxidizes myo-inositol to glucuronic acid^17,18^. High levels of serum MIOX has been associated with AKI, and levels of serum MIOX increased even before the rise in serum creatinine suggestive of markers for detection of AKI^11,12^. A recent study investigated the effectiveness of MIOX in the early diagnosis in ischemia–reperfusion mice models and found increased serum MIOX levels in the early stages of AKI^19^. In another study, overexpression of MIOX led to worsening of AKI in cisplatin-induced injury, and disruption in MIOX gene seemed to be protective against cisplatin-induced AKI^20^, Further, in type 2 diabetic nephropathy (T2DN), expression of MIOX correlates with tubulointerstitial damage^21^ A few studies have explored pathways that might explain the mechanism of the role of MIOX in AKI-CKD progression. In vitro overexpression of MIOX triggers production of inflammatory cytokines and generation of reactive oxygen species^21^. Tominaga et al.^22^ showed that MIOX-induced oxidative stress linked to tunicamycin-induced endoplasmic reticulum stress may step-up the tubulointerstitial injury. Sharma et al.^23^ showed that gentamycin induced cellular injury in the AKI is further exacerbated by the overexpression of MIOX. Further, MIOX-mediated redox injury and necroptosis are involved in the pathogenesis of cadmium-induced renal injury^24^. Another study showed that ferroptosis is modulated by MIOX overexpression in cisplatin-induced AKI^25^. There are no data, however, on the predictive ability of MIOX for progression to acute kidney disease (AKD) or CKD. In the current study, serum MIOX showed an AUC of 0.85, which suggest that serum MIOX levels can be used to identify patients for whom a closer follow-up after discharge will be needed, as AUC above 0.80 is strongly considered clinically useful^26^.

In the past, levels of urine and or serum NGAL, KIM and LFABP1 have been explored as a early predictive biomarkers for renal recovery from AKI. Out of these, NGAL has been recognized as a potential candidate biomarker for outcome of AKI in recent reports^27–30^ and has received the most attention. In our study high urinary NGAL was independently associated with progression to CKD from CA-AKI in univariate and multivariable logistic regression analysis. These results are similar to the data reported by Srisawat et al., in 181 patients with community acquired pneumonia and AKI. High plasma NGAL level at first day of diagnosis by Risk, Injury, Failure, Loss, and End-stage kidney disease (RIFLE-F) classification, predicted failure to recovery of renal function. Recovery of renal function was defined as being alive at discharge and KRT independence during hospitalization and no persistent RIFLE-F at hospital discharge (AUC 0.71)^29^. In another study on 76 critically ill AKI patient requiring KRT, the decrease of urinary NGAL from day 1 to day 14 predicted renal recovery (AUC 0.70) defined as being alive and without requirement for dialysis by day 60^28^. In the study by Moon et in 66 AKI patients, low urinary NGAL at day 0 was able to predict renal recovery (AUC 0.78) (50% or more decrease in plasma creatinine from the maximum level)^27^. Another study on 145 hospital acquired AKI, elevated levels of urinary NGAL at the time of AKI diagnosis predicted poor long term (6 months) outcome (new onset of end stage kidney disease or all-cause mortality)^31^.

The timing of biomarker analysis is an important consideration while looking at the value of biomarkers as a predictive tool in AKI survivors. Currently, there is no standardized or appropriate timepoint to measure the biomarkers for prediction of recovery form AKI. The timing of biomarker analysis for prediction of recovery depends on the context of AKI. Levels of biomarkers may be affected by the stages or severity of AKI and may not be accurate in predicting the long-term renal outcome. We investigated the predictive ability of biomarkers at the time of discharge from hospital, as risk stratification is of greatest value when performed at the time of discharge to develop plans for follow up and monitoring^32^. This allows efficient utilization of resources and reduces waste. Also, we used penalized LASSO logistic regression model to assess the predicting ability of the biomarkers for progression to CKD. As compared to traditional methods, penalised regression methods are preferred when there are far more variables than observations which in turn could raise the issue of multicollinearity and overfitting. This results in shrinking the coefficients of the less contributive variables toward zero and thus removing them from final multivariable model. Another strength of this study is that it is a unique cohort of CA-AKI patients selected with stringent inclusion and exclusion criteria with a complete 4 month follow up.

A few limitations of our study need to be acknowledged, however. They include its single-center observational nature, small sample size; relatively short follow up, and the possibility of an effect of kidney function on the biomarker levels. Most of the patients with AKI were in advanced stage of AKI. Further, this analysis has not been validated in an external cohort.

In conclusion, serum level of MIOX at the hospital discharge could be used as predictive biomarker for renal recovery from community acquired AKI either alone or in multivariable model including serum creatinine and uPCR. The results of this study need validation in larger population of AKI with longer follow up to establish this association and utility of this model in clinical decision making for further stratification of patients at the risk of developing CKD.

## Materials and methods

### Study setting

This study was a prospective, observational cohort study in patients with CA-AKI conducted in a tertiary care hospital Postgraduate Institute of Medical Education & Research (PGIMER), Chandigarh, India.

### Study population

The study was conducted between February 2017 and January 2020. All subjects between the ages of 18 and 70 years admitted at PGIMER and diagnosed to have AKI at any time during hospital stay and discharged from hospital were eligible for screening. CA-AKI was defined as established AKI at the time of admission or AKI developed withing 48 h of initial contact with health care services. Patients with pre-existing CKD, and a diagnosis of glomerulonephritis, obstructive uropathy, malignancies, decompensated chronic liver disease, heart failure, or who had received solid-organ or bone marrow transplants were excluded. Lastly, patients who had a high likelihood of re-hospitalization or death in the following 4 months were also excluded. The study was approved by Institute Ethics Committee of PGIMER, Chandigarh, India, and informed consent was obtained from each patient before enrolment in this study. All experiments were performed in accordance with relevant guidelines and regulations.

### Study procedure and follow-up

Baseline characteristics including demographic details, comorbidities, markers of severity of AKI including dialysis details, and laboratory data were collected. Blood and urine samples were collected at the time of discharge from hospital and stored at − 80 °C for analysis of serum and urine biomarkers MIOX and YAP, urine NGAL, KIM1, L-FABP, urine quinolinate and tryptophan levels and spot urine protein creatinine ratio (uPCR). The patients were followed up at 4 months after hospital discharge for assessment of renal outcome.

### Measurement of biomarkers

Levels of MIOX and YAP in serum were measured using ELISA kits by USCN Life Science Inc., Wuhan, Hubei, China, and MyBioSource Inc., San Diego, CA, USA, respectively. MIOX and YAP concentrations in urine samples were measured using ELISA kits by Bioassay Technology Laboratory, Yangpu, Shanghai, China. Urinary NGAL and KIM-1 were measured by Quantikine® ELISA kits (R&D Systems, Inc., Minneapolis, MN, USA). Urinary L-FABP, was measured using ELISA kit by Elabscience, Houston, TX, USA.

Measurement of quinolinate and tryptophan in spot urine samples was done by high-performance liquid chromatography (HPLC) method using UltiMate 3000 UHPLC (Thermo Fisher Scientific Inc., Waltham, MA USA). Samples were thawed and 300 μL sample was taken in a 1.8 mL tube. Samples were then vortexed strongly for 1 min. 300 μL of 100% ethanol was then added to the sample followed by strong vortex for 30 s. The samples were then incubated at − 20 °C for 20 min followed by centrifugation at 12,000×*g* for 20 min at 4 °C. Finally, 100 μL of supernatant was taken carefully and transferred to new vials and 20 μL of volume was injected for HPLC analysis under the following conditions: flow rate: 1 mL/min; injection volume: 20 μL; UV HPLC range: 210 nm; running time: 10 min; machine temperature: 30 °C. Standards for tryptophan and quinolinate were also run along with the samples and were used for analyse the levels of tryptophan and quinolinate in urine samples.

### Study outcome

Study outcome was progression to CKD from CA-AKI at 4 months after hospital discharge. Renal recovery was defined as eGFR (CKD EPI-2009) > 60 ml/min/1.73 m^2^ and spot urine protein to creatinine ratio (uPCR) < 500 mg/g. Patients who had eGFR < 60 ml/min/1.73 m^2^ or were dialysis dependent at 4 month after discharge were defined as having progressed to CKD.

### Statistical considerations

Descriptive statistics was used to describe the baseline demographic and clinical characteristics of study subjects. Continuous data were presented as mean ± standard deviation (SD) or median (25th, 75th percentile), and categorical data were presented as frequency (percentage). To compare continuous data, student t-test or Mann–Whitney *U* test was performed. Categorical data was compared using the chi-square or Fisher exact test. Association between CKD progression and each biomarker was assessed separately using logistic regression. Adjusted logistic regression was used to study whether biomarker is independently associated with progression to CKD, after adjusting for other clinical parameters and demographic variables. Penalized least absolute shrinkage and selection operator (LASSO) logistic regression model was performed on all serum and urine biomarkers to do variable selection. Finally, multivariable logistic regression was fit for only select variables obtained from penalized LASSO model to assess their association with progression to CKD. Receiver operating characteristic (ROC) curves were plotted for multivariable model including multiple biomarkers in addition to ROC curves for the individual biomarkers. The area under the curve (AUC) values at 95% CI were reported for the multivariable logistic regression model and individual biomarkers. Non-normally distributed continuous variables namely, serum MIOX (pg/ml), serum YAP (pg/ml), urine MIOX/creatinine (pg/g), urine YAP/creatinine (ng/g), urine KIM-1/creatinine (pg/g), urine NGAL/creatinine (ng/g), urine l-FABP/creatinine (ng/g), urine quinolinate/tryptophan (Q/T), uPCR (mg/m), discharge creatinine was standardized log transformed for logistic regression analysis. All P values were two-tailed and considered significant when < 0.05. All analyses were performed using STATA version 22 (IBM Corp., Armonk, NY, USA).

### Ethics approval and consent to participate

Study was approved from the Institute Ethics Committee and patients were enrolled in this study after getting the written prior to study procedures.

### Consent for publication

Consent form available on request.

## Supplementary Information

Supplementary Table S1.

## Data Availability

The data which are presented in this study are available with the corresponding author.
