# Supplementary material for: Serum myo-inositol oxygenase levels at hospital discharge predict progression to chronic kidney disease in community-acquired acute kidney injury
Source: Sci Rep. 2022 Aug 2;12:13225. doi: 10.1038/s41598-022-17599-w (PMC9345942; doi:10.1038/s41598-022-17599-w)
Supplement: Supplementary file 1 — Supplementary Table S1. [file 41598_2022_17599_MOESM1_ESM.pdf]

Serum myo-inositol oxygenase levels at hospital discharge predict progression to chronic kidney disease in community-acquired acute kidney injury

Tom Jose Kakkanattu[1*], Jaskiran Kaur[2*], Vinod Nagesh[1], Monica Kundu[3], Kajal Kamboj[1], Prabhjot Kaur[1], Jasmine Sethi[1], Harbir Singh Kohli[1], Kishan Lal Gupta[1], Arpita Ghosh[3], Vivek Kumar[1], Ashok Kumar Yadav[2], Vivekanand Jha[3,4,5]

[1]Department of Nephrology, Postgraduate Institute of Medical Institute Education and Research, Chandigarh, India; [2]Department of Experimental Medicine and Biotechnology, Postgraduate Institute of Medical Institute Education and Research, Chandigarh, India; [3]George Institute for Global Health, UNSW, New Delhi, India; [4]School of Public Health, Imperial College, London, UK; [5]Manipal Academy of Higher Education, Manipal, India

Table S1: Coefficient for variable selected using Penalized LASSO logistic regression

Variables	Coefficients
Intercept	-1.857
Male®<br>Female	 -0.409
Age	0.000
Hypertension	0.769
Hospital duration	0.015
Serum MIOX	0.506
Spot uPCR	0.431
Discharge serum creatinine	0.403
Model accuracy	89%

Multivariable logistic model derived using penalised regression, with model coefficients shown.

To use the model, individual patient values could be entered into the following equation to derive a linear predictor (LP) of non-recovery (outcome):

Linear predictor = -1.857+ (female * -0.409) + (age *0.000) + (hypertension*0.769) + (hospital duration*0.015) +(serum miox * 0.506) + (spot pcr * 0.431) + discharge creatinine * 0.403.
